# Association between adherence to and persistence with atypical antipsychotics and psychiatric relapse among US Medicaid-enrolled patients with schizophrenia

**DOI:** 10.1111/jphs.12004

**Published:** 2013-01-07

**Authors:** Jessica Panish, Sudeep Karve, Sean D Candrilli, Riad Dirani

**Affiliations:** a Janssen Scientific Affairs, LLCTitusville, New Jersey, USA; b RTI Health SolutionsResearch Triangle Park, North Carolina, USA

**Keywords:** adherence, atypical antipsychotics, persistence, schizophrenia

## Abstract

**Objective:**

Assess association between adherence and persistence with second-generation oral antipsychotics (SGOAs), psychiatric-related relapse and healthcare utilization among patients with schizophrenia experiencing two or more psychiatric-related relapses.

**Methods:**

A retrospective analysis of the US Medicaid Multi-State Database for 2004–2008. Patients with schizophrenia (aged 18–64) with two or more psychiatric-related relapses within 1 year after SGOA initiation were selected. Associations between a dichotomous measure of adherence and persistence with SGOAs and psychiatric-related relapse and healthcare utilization were assessed using unadjusted and covariate-adjusted regression models. No adjustment was made for multiplicity.

**Key findings:**

Study cohort consisted of 3714 patients with mean age of 42.6 years. Overall, 45% of patients were adherent and 50% persistent with SGOAs. Unadjusted and covariate-adjusted analysis results suggested the 12-month psychiatric-related relapse rate was lower among adherent/persistent patients versus non-adherent patients (unadjusted mean: 3.85 versus 4.13; *P* < 0.001; covariate-adjusted incident rate ratio (IRR): 0.90; 95% confidence interval (CI): 0.86–0.94) and non-persistent patients (unadjusted mean: 3.81 versus 4.21; *P* < 0.001; covariate-adjusted IRR: 0.88; 95%CI: 0.84–0.92). Compared with non-persistent patients, persistent patients had significantly lower rates of all-cause inpatient admissions (IRR: 0.87; 95%CI: 0.82–0.93) and emergency department visits (IRR: 0.78; 95%CI: 0.73–0.85).

**Conclusions:**

Although SGOAs have proven efficacy in lowering the rate of psychiatric-related relapses, lower adherence and persistence rates may be an inhibiting factor in achieving optimal benefits from SGOAs. Future research is needed to assess whether newer antipsychotics with less-frequent dosing may improve adherence among patients with schizophrenia.

## Introduction

Schizophrenia is a complex chronic psychiatric disorder characterized by psychosis, hallucinations, delusions, disorganized speech and behaviour, cognitive deficits, reduced emotional expression and social withdrawal. Patients with schizophrenia typically require lifelong antipsychotic use for disease management and relapse control. However, compared with earlier-developed conventional antipsychotics, second-generation oral antipsychotics (SGOAs) are the preferred choice of physicians in the management of schizophrenia, mainly because of the lower rate of extrapyramidal adverse events (AEs). Several clinical trials have effectively established the efficacy of SGOAs for maintenance treatment of schizophrenia.[1–4]

For chronic conditions, like schizophrenia, that require lifelong medication use, adherence is an important factor in achieving benefits of the prescribed therapy.[5,6] Recent survey findings indicated that physicians observed medication adherence rates of 51–70% in their practices.[7] Non-adherence to the prescribed antipsychotic therapy is associated with increased risk of relapse,[8–10] greater healthcare utilization[11,12] and higher costs.[13–15] A study by Weiden and Olfson[10] reported that non-adherence to antipsychotic therapy accounted for approximately 40% of re-hospitalizations among patients with schizophrenia.[10] Similarly, a recent literature review of inpatient costs related to antipsychotic non-adherence in the USA estimated that re-hospitalizations costs associated with antipsychotic non-adherence were US$1.48 billion (range, US$1.39–1.83 billion) in 2005.[14]

In addition to adherence, persistence with the prescribed medication, which is defined as ‘the duration of time from initiation to discontinuation of therapy’ is considered an important factor in managing chronic conditions like schizophrenia.[5,16] Several studies have compared persistence with various individual conventional and atypical antipsychotic medications among patients with schizophrenia.[17–20] For example, a study evaluating persistence with conventional and atypical antipsychotic therapy among patients with schizophrenia who are enrolled in the US Medicaid health services programme reported that 58% of patients initiating conventional antipsychotic agent discontinued the medication compared with 33% for patients initiating atypical antipsychotics.[20] Even though studies have assessed persistence with antipsychotic therapy, limited data exist assessing the impact of non-persistence on the healthcare utilization and costs among patients with schizophrenia. A study conducted by Weiden *et al*. reported that lower rates of antipsychotic therapy persistence were associated with higher rates of psychiatric-related hospitalization.[9]

Although studies evaluated the association between antipsychotic therapy adherence, persistence, healthcare utilization and costs, to the best of the authors' knowledge no studies have assessed this association among patients experiencing multiple psychiatric-related relapses while receiving SGOAs. Prior research suggests that patients with schizophrenia experiencing a psychiatric-related relapse event are significantly more likely to experience a second event with the first event being the most significant predictor of the second relapse event.[13] Moreover, patients experiencing multiple psychiatric-related relapses incur five times higher healthcare costs than patients without relapse.[12] Understanding the association between adherence to and persistence with SGOAs and healthcare utilization among patients with multiple relapse events is important. With the objective of addressing the current literature gap, our study examined the association between adherence to and persistence with SGOAs, psychiatric-related relapses and healthcare utilization among Medicaid-enrolled patients with schizophrenia. Since Medicaid serves as the largest payer for mental-health care in the USA, we limited our study to Medicaid-enrolled patients with schizophrenia.[21]

## Methods

### Data source and patient selection

We conducted a retrospective longitudinal analysis of the Thomson Reuters' MarketScan Medicaid Multi-State Database for 2004–2008. Data for >26 million Medicaid enrollees from 11 states are represented in this database; for confidentiality purposes, names of states are not reported.

The data include medical and pharmacy (excluding drugs provided in an inpatient setting) utilization records and enrolment and demographic (e.g. age, race, gender) information for Medicaid enrollees A unique, encrypted identification number was available for each enrollee, enabling the longitudinal tracking of individuals' healthcare utilization and costs over time. All elements included in these data are in accordance with the US Health Insurance Portability and Accountability Act definition for limited-use datasets.[22] The institutional review board at RTI International, New Carolina, USA, approved the conduct of this study.

We initially selected adults (≥18 years of age) with ≥1 claim for any SGOA during 1 January 2005 to 31 December 2007. The date corresponding to the first evidence of SGOA prescription claim defined the ‘index SGOA date.’ The index SGOA date served as the reference date to define the ‘preindex’ (i.e. 12-month period before the index SGOA date) and ‘postindex’ date periods (i.e. 12-month period on or after the index SGOA date). All patients were required to have ≥2 schizophrenia diagnoses (*International Classification of Diseases,* 9th Revision, Clinical Modification (ICD-9-CM) code 295.xx) in the medical records during the preindex date period. Moreover, patients were required to have ≥2 psychiatric relapse events during the postindex period. Patients with an inpatient admission or emergency department (ED) visit for psychiatric conditions (ICD-9-CM codes available upon request) were defined as having a psychiatric-related relapse event. Patients were required to have continuous Medicaid enrolment during the study periods. Finally, patients' ≥65 years old at any point during the observation period or patients with dual coverage (i.e. both Medicaid and Medicare) were excluded.

### Adherence and persistence with second-generation oral antipsychotics

#### Adherence to SGOAs

Postindex period adherence to SGOAs was measured using the adherence measure medication possession ratio (MPR), defined as:


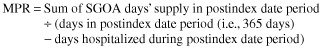


Because the data did not provide details on inpatient drug use, the number of days a patient was hospitalized during the postindex date period was subtracted from the denominator, assuming that patients received, and took as prescribed, their SGOA during hospitalization.

#### Persistence with SGOAs

Persistence with SGOAs was evaluated by documenting the distribution of patients who had ≥1 therapy interruption (i.e. refill gap of ≥60 days) with subsequent re-initiation, or discontinuation of therapy without subsequent re-initiation, or continuous use during the postindex date period.[23] Three categories were used to describe persistence: category 1: therapy interruption (a refill gap of ≥60 days, with subsequent re-initiation); category 2: therapy discontinuation (without subsequent SGOA re-initiation); category 3: continuous use (remaining on the index SGOA or switching to a different SGOA within the permissible gap of <60 days).

### Study outcomes

The primary outcomes assessed included overall (all-cause), schizophrenia-related healthcare utilization, and psychiatric-related relapses during the postindex date period. To identify schizophrenia-related medical and pharmacy utilization, medical claims with a schizophrenia diagnosis and pharmacy claims for first- or second-generation antipsychotics were used. All-cause and schizophrenia-related healthcare utilizations were reported for the following categories: (1) the percentage of patients with ≥1 claim for the selected categories of service; (2) the number of unique inpatient and ED admissions; and (3) the number of physician office, outpatient and other ancillary care visits and pharmacy claims.

Finally, we documented the number of psychiatric-related relapses during the postindex date period.

### Covariates

The primary independent variables for this study included binary indicators for SGOA adherence and persistence. Using a previously validated adherence threshold value (i.e. 80%), we classified patients in to ‘non-adherent’ (MPR < 0.80) and ‘adherent’ (MPR ≥ 0.80) categories.[24] A dichotomous measure of persistence was also created; patients with SGOA interruption or discontinuation were categorized as ‘non-persistent’ and patients with continuous SGOA use were categorized as ‘persistent.’ Other covariates considered for this analysis include age, gender, race/ethnicity health plan type, basis of Medicaid eligibility, mental-health substance abuse coverage, dose escalation, polypharmacy and comorbidity burden assessed using the Charlson Comorbidity Index score.[25,26]

### Statistical analysis

All statistical analyses were conducted using SAS (version 9.1.3) statistical software package (SAS Institute Inc., Cary, North Carolina, USA). All analyses were stratified by adherence and persistence status. Descriptive statistics were generated, including frequencies for categorical variables and mean values and standard deviations (SDs) for continuous variables. Both unadjusted univariate and covariate-adjusted multivariable analyses were conducted to assess differences in outcomes by adherence and persistence status. Univariate analyses included assessing differences in baseline demographic characteristics and outcome measures, including all-cause and schizophrenia-related healthcare utilization and psychiatric-related relapses using Student *t*-test (for continuous variables) and the chi-squared or Fisher exact test (for categorical variables).

Multivariable regression analyses were performed to assess differences in all-cause and schizophrenia-related healthcare utilization and psychiatric-related relapses between the adherent and persistent groups, after adjusting for demographic characteristics and baseline comorbidity burden. Two separate multivariable regression models were estimated for each outcome measure, one with a binary indicator for adherence and another with a binary indicator for persistence. Multivariable negative binomial and Poisson regression analyses were performed for outcomes categorized as count data (e.g. number of psychiatric-related relapses). For all count outcome measures, negative binomial regression models were ultimately deemed a better fit based on goodness-of-fit tests (assessed using Pearson chi-squared statistic) conducted for each model estimated. Incident rate ratios (IRRs) were obtained by exponentiating the coefficients from each model estimated. The magnitude of IRRs describes the rate of utilization between adherent (or persistent) patients versus non-adherent (or non-persistent) patients.

For each dichotomous outcome measure, a logistic regression model was estimated. Odds ratios (ORs) were obtained by exponentiating the coefficients from each logistic regression model estimated. Odds ratios illustrate the increased or decreased likelihood of event in adherent (or persistent) versus non-adherent (or non-persistent) patients. No adjustment was made for multiplicity.

## Results

The final study sample consisted of 3714 patients who met all study inclusion and exclusion criteria.

### Therapy adherence and persistence with second-generation oral antipsychotics

Table 1 presents a descriptive summary of adherence to and persistence with SGOAs during the postindex date period. The mean (standard deviation (SD)) medication possession ratio (MPR) of the study cohort was 0.66 (0.30). Approximately 45% of schizophrenia patients were adherent (MPR ≥ 0.80) to SGOAs. The mean MPR for the adherent and non-adherent groups was 0.94 (0.06) and 0.43 (0.22), respectively. Approximately 50% of schizophrenia patients were persistent with SGOAs. Among patients non-persistent with SGOAs, 23% had ≥1 therapy interruption of ≥60 days, whereas 27% discontinued SGOAs without subsequent re-initiation.

**Table 1:** Adherence to and persistence with second-generation oral antipsychotic therapy among US Medicaid-enrolled patients with schizophrenia

Characteristics	All patients
Total sample size, *n* (%)	3714 (100.00)
Adherence^a^	
MPR, mean (SD)^b^	0.66 (0.30)
MPR distribution (0.80 threshold), *n* (%)	
0–0.19	415 (11.17)
0.20–0.39	491 (13.22)
0.40–0.59	565 (15.21)
0.60–0.79	570 (15.35)
0.80–1.00	1673 (45.05)
Non-adherent total (MPR < 0.80), *n* (%)	2041 (54.95)
Adherent total (MPR ≥ 0.80), *n* (%)	1673 (45.05)
Persistence, *n* (%)^c^	
Therapy interruption (refill gap > 60 days with subsequent re-initiation)	848 (22.83)
Therapy discontinuation (without subsequent SGOA re-initiation)	1010 (27.19)
Continuous SGOA use	1856 (49.97)
Non-persistent total (had therapy interruption or discontinuation)	1858 (50.03)
Persistent total (had continuous SGOA use)	1856 (49.97)

a Adherence measured during the 12-month postindex period after the first evidence of SGOA use.

b MPR, total days of SGOA therapy ÷ (365 – days hospitalized).

c Persistence measured during the 12-month postindex period after the first evidence of SGOA use. Persistence is defined as continuous use of SGOAs (i.e. no refill gap or refill gap of ≤60 days for SGOA medication). Non-persistence is defined as therapy interruption (i.e. refill gap for the SGOA medication > 60 days) or SGOA discontinuation.

MPR, medication possession ratio; SD, standard deviation; SGOA, second-generation oral antipsychotic.

### Demographic characteristics

Table 2 presents descriptive statistics on various baseline demographic characteristics of the study sample, both overall and by SGOA adherence and persistence status.

**Table 2:** Baseline characteristics, by adherence and persistence status

Characteristic	All patients	Adherent^a^	Non-adherent	*P* value^c^	Persistent^a^	Non-persistent	*P* value^c^
Total sample, *n* (%)	3714 (100.00)	1673 (100.00)	2041 (100.00)	–	1856 (100.00)	1858 (100.00)	–
Gender, *n* (%)							
Male	1641 (44.18)	683 (40.82)	958 (46.94)	<0.001	762 (41.06)	879 (47.31)	<0.001
Female	2073 (55.82)	990 (59.18)	1083 (53.06)	<0.001	1094 (58.94)	979 (52.69)	<0.001
Age at index date^c^							
Mean (SD)	42.62 (11.63)	43.44 (12.11)	41.94 (11.19)	<0.001	43.50 (11.84)	41.74 (11.36)	<0.001
Median	44.00	45.00	43.00		45.00	43.00	
Age distribution, y, *n* (%)							
18–24	316 (8.51)	146 (8.73)	170 (8.33)	0.666	143 (7.70)	173 (9.31)	0.079
25–34	674 (18.15)	277 (16.56)	397 (19.45)	0.023	317 (17.08)	357 (19.21)	0.092
35–44	924 (24.88)	383 (22.89)	541 (26.51)	0.011	442 (23.81)	482 (25.94)	0.134
45–54	1196 (32.20)	527 (31.50)	669 (32.78)	0.407	592 (31.90)	604 (32.51)	0.690
55–64	604 (16.26)	340 (20.32)	264 (12.93)	<0.001	362 (19.50)	242 (13.02)	<0.001
Race/ethnicity, *n* (%)							
White	1628 (43.83)	931 (55.65)	697 (34.15)	<0.001	989 (53.29)	639 (34.39)	<0.001
African American	1799 (48.44)	622 (37.18)	1177 (57.67)	<0.001	727 (39.17)	1072 (57.70)	<0.001
Hispanic	21 (0.57)	12 (0.72)	9 (0.44)	0.264	13 (0.70)	8 (0.43)	0.273
Other	266 (7.16)	108 (6.46)	158 (7.74)	0.131	127 (6.84)	139 (7.48)	0.451
Health coverage, *n* (%)							
Fee-for-service	2529 (68.09)	1185 (70.83)	1344 (65.85)	0.001	1289 (69.45)	1240 (66.74)	0.076
Capitated	1185 (31.91)	488 (29.17)	697 (34.15)	0.001	567 (30.55)	618 (33.26)	0.076
Mental-health substance abuse, *n* (%)							
Yes	3607 (97.12)	1623 (97.01)	1984 (97.21)	0.723	1801 (97.04)	1806 (97.20)	0.764
No	107 (2.88)	50 (2.99)	57 (2.79)	0.723	55 (2.96)	52 (2.80)	0.764
Basis of Medicaid eligibility, *n* (%)							
Blind/disabled individual	3585 (96.53)	1618 (96.71)	1967 (96.37)	0.576	1796 (96.77)	1789 (96.29)	0.424
Adult (not based on unemployed status)	60 (1.62)	16 (0.96)	44 (2.16)	0.004	20 (1.08)	40 (2.15)	0.009
Child (not child of unemployed adult, not foster care child)	44 (1.18)	23 (1.37)	21 (1.03)	0.332	26 (1.40)	18 (0.97)	0.224
Other^d^	25 (0.67)	16 (0.96)	9 (0.44)	0.056	14 (0.75)	11 (0.59)	0.545
CCI score (preindex date period)							
Mean (SD)	1.62 (2.18)	1.75 (2.19)	1.51 (2.17)	0.001	1.71 (2.15)	1.53 (2.20)	0.011
Median	1.00	1.00	1.00		1.00	1.00	

a Adherent (MPR ≥ 80%) and non-adherent (MPR < 80%). Persistence was defined as continuous use of SGOAs or a refill gap for the SGOA medication ≤ 60 days. Non-persistence was defined as a therapy interruption (i.e. refill gap for the SGOA medication > 60 days) or SGOA discontinuation.

b *P* values based on Student's *t* test for continuous variables and chi-squared or Fisher test for categorical variables.

c The date corresponding to the first observed SGOA prescription claim during the period 1 January 2005 to 31 December 2007.

d Other includes foster-care child, aged individual and eligibility status unknown.

CCI, Charlson Comorbidity Index; MPR, medication possession ratio; SD, standard deviation; SGOA, second-generation oral antipsychotic.

The mean age of the study cohort was 42.62 (11.63) years, with adherent (43.44 (12.11) years) and persistent (43.50 (11.84) years) patients being significantly older than non-adherent (41.94 (11.19) years; *P* < 0.001) and non-persistent (41.74 (11.36) years; *P* < 0.001) patients. Approximately 56% of the population was female. African Americans accounted for over 48% of the study cohort and were significantly less likely to be adherent to (*P* < 0.001) and persistent with (*P* < 0*.*001) SGOAs, when compared with other racial groups. Approximately 97% of patients had mental-health substance coverage during the follow-up period. Higher percentage of adherent (70.8%) and persistent (69.4%) patients had fee-for-service health coverage compared with the non-adherent (65.8%) and non-persistent (66.7%) patients. Across both adherence and persistence groups over 96% of the patients had ‘blind/disabled individual’ listed as the reason for Medicaid eligibility. The adherent and persistent patients had significantly higher comorbidity burdens than non-adherent (*P* *=* 0*.*001) and non-persistent (*P* *=* 0*.*011) patients.

### Unadjusted association between second-generation oral antipsychotics adherence and persistence and psychiatric-related relapses as well as schizophrenia-related and all-cause utilization

Table 3 presents a descriptive summary of psychiatric-related relapses and schizophrenia-related and all-cause healthcare utilization, by adherence and persistence status. During the postindex date period, the mean (SD) number of psychiatric-related relapses experienced by schizophrenia patients was 4.01 (3.34). The mean (SD) number of psychiatric-related relapses differed significantly among adherent (3.85 (3.28)) versus non-adherent (4.13 (3.37); *P* *=* 0*.*012) and persistent (3.8 (3.20)) versus non-persistent (4.21 (3.46); *P* < 0*.*001) schizophrenia patients. Schizophrenia patients had a mean (SD) 1.20 (1.80) schizophrenia-related inpatient stays during the postindex date period, with no significant differences in mean number of inpatient stays observed by patient adherence or persistence status. Patients persistent with the SGOA therapy had lower mean schizophrenia-related inpatient stay compared with the non-persistent patients (18.15 (19.88) days versus 21.43 (31.82) days; *P* *=* 0.006). The schizophrenia-related inpatient stay did not differ by adherence status. A significantly smaller proportion of patients adherent to and persistent with SGOAs had an ED visit during the postindex date period, compared with non-adherent (29.65% versus 35.13%; *P* < 0*.*001) and non-persistent (30.44% versus 34.88%; *P* *=* 0*.*004) patients. Both patients adherent to and persistent with SGOA therapy had higher mean number of schizophrenia-related office visits compared with non-adherent (11.22 (30.68) versus 5.02 (16.90); *P* < 0.001) and non-persistent (10.35 (28.67) versus 5.28 (18.60); *P* < 0.001) patients. Similarly, schizophrenia-related outpatient visits were higher among adherent and persistent groups compared with the non-adherent (1.50 (4.02) versus 1.09 (3.10); *P* < 0.001) and non-persistent patients (1.45 (3.90) versus 1.11 (3.15); *P* *=* 0.004).

**Table 3:** Summary of healthcare utilization during the 12-month period after first evidence of second-generation oral antipsychotic therapy use, by adherence and persistence status

	All patients	Adherent^a^	Non- adherent	*P* value^b^	Persistent^a^	Non-persistent	*P* value^c^
Psychiatric-related relapse events^c^							
Number of relapse events							
Mean (SD)	4.01 (3.34)	3.85 (3.28)	4.13 (3.37)	0.012	3.81 (3.20)	4.21 (3.46)	<0.001
Median	3.00	3.00	3.00		3.00	3.00	
All-cause and schizophrenia-related healthcare utilization							
Had ≥1 encounter, *n* (%)	3714 (100.00)	1673 (100.00)	2041 (100.00)	–	1856 (100.00)	1858 (100.00)	–
Had ≥1 schizophrenia-related encounter, *n* (%)	3714 (100.00)	1673 (100.00)	2041 (100.00)	–	1856 (100.00)	1858 (100.00)	–
Number of encounters, mean (SD)	153.14 (105.65)	204.71 (109.84)	110.86 (80.36)	<0.001	194.75 (108.77)	111.57 (83.91)	<0.001
Number of schizophrenia-related encounters, mean (SD)	55.55 (60.27)	77.16 (70.65)	37.83 (42.69)	<0.001	72.59 (67.39)	38.53 (46.33)	<0.001
Had ≥1 medical (i.e. non-pharmacy) encounter, *n* (%)	3714 (100.00)	1673 (100.00)	2041 (100.00)	–	1856 (100.00)	1858 (100.00)	–
Had ≥1 schizophrenia-related medical (i.e. non-pharmacy) encounter, *n* (%)	3449 (92.86)	1568 (93.72)	1881 (92.16)	0.066	1736 (93.53)	1713 (92.20)	0.113
Number of medical encounters, mean (SD)	78.53 (67.31)	100.18 (78.00)	60.79 (50.59)	<0.001	95.26 (75.40)	61.82 (53.10)	<0.001
Number of schizophrenia-related medical encounters, mean (SD)	39.12 (55.37)	53.08 (67.20)	27.67 (39.84)	<0.001	49.77 (63.76)	28.47 (42.92)	<0.001
Had ≥1 pharmacy claim, *n* (%)	3714 (100.00)	1673 (100.00)	2041 (100.00)	–	1856 (100.00)	1858 (100.00)	–
Had ≥1 schizophrenia-related pharmacy claim, *n* (%)	3714 (100.00)	1673 (100.00)	2041 (100.00)	–	1856 (100.00)	1858 (100.00)	–
Number of pharmacy claims, mean (SD)	74.61 (59.32)	104.53 (61.87)	50.08 (43.94)	<0.001	99.50 (61.58)	49.75 (44.83)	0<.001
Number of schizophrenia-related pharmacy claims, mean (SD)	16.43 (12.65)	24.09 (13.41)	10.16 (7.52)	<0.001	22.82 (13.15)	10.06 (8.11)	<0.001
Had ≥1 ED visit, *n* (%)	3231 (87.00)	1408 (84.16)	1823 (89.32)	<0.001	1581 (85.18)	1650 (88.81)	0.001
Had ≥1 schizophrenia-related ED visit, *n* (%)	1213 (32.66)	496 (29.65)	717 (35.13)	<0.001	565 (30.44)	648 (34.88)	0.004
Number of ED visits, mean (SD)	5.35 (7.79)	4.35 (6.36)	6.16 (8.71)	<0.001	4.61 (6.70)	6.08 (8.68)	<0.001
Number of schizophrenia-related ED visits, mean (SD)	0.59 (1.30)	0.49 (1.05)	0.68 (1.47)	<0.001	0.50 (1.04)	0.68 (1.50)	<0.001
Had ≥1 office visit, *n* (%)	3232 (87.02)	1531 (91.51)	1701 (83.34)	<0.001	1700 (91.59)	1532 (82.45)	<0.001
Had ≥1 schizophrenia-related office visit, *n* (%)	1329 (35.78)	688 (41.12)	641 (31.41)	<0.001	738 (39.76)	591 (31.81)	<0.001
Number of office visits, mean (SD)	17.02 (28.58)	21.95 (34.22)	12.97 (22.14)	<0.001	21.18 (33.47)	12.86 (21.89)	<0.001
Number of schizophrenia-related office visits, mean (SD)	7.81 (24.29)	11.22 (30.68)	5.02 (16.90)	<0.001	10.35 (28.67)	5.28 (18.60)	<0.001
Had ≥1 outpatient visit, *n* (%)	3005 (80.91)	1455 (86.97)	1550 (75.94)	<0.001	1601 (86.26)	1404 (75.57)	<0.001
Had ≥1 schizophrenia-related outpatient visit, *n* (%)	1229 (33.09)	608 (36.34)	621 (30.43)	0.001	668 (35.99)	561 (30.19)	<0.001
Number of outpatient visits, mean (SD)	6.21 (9.63)	7.42 (11.55)	5.23 (7.57)	<0.001	7.19 (11.20)	5.24 (7.64)	<0.001
Number of schizophrenia-related outpatient visits, mean (SD)	1.28 (3.55)	1.50 (4.02)	1.09 (3.10)	<0.001	1.45 (3.90)	1.11 (3.15)	0.004
Had ≥1 inpatient stay, *n* (%)	3066 (82.55)	1409 (84.22)	1657 (81.19)	0.015	1536 (82.76)	1530 (82.35)	0.741
Had ≥1 schizophrenia-related inpatient stay, *n* (%)	2040 (54.93)	912 (54.51)	1128 (55.27)	0.646	993 (53.50)	1047 (56.35)	0.081
Number of inpatient stays, mean (SD)	2.42 (2.67)	2.50 (2.86)	2.35 (2.50)	0.092	2.36 (2.73)	2.48 (2.60)	0.202
Length of stay,^d^ mean (SD)	22.73 (29.12)	24.00 (28.96)	21.65 (29.21)	0.026	20.58 (23.12)	24.89 (33.96)	<0.001
Number of schizophrenia-related inpatient stays, mean (SD)	1.20 (1.80)	1.25 (2.05)	1.17 (1.57)	0.206	1.18 (1.94)	1.23 (1.65)	0.332
Schizophrenia-related length of stay,^d^ mean (SD)	19.83 (26.73)	20.92 (25.25)	18.95 (27.85)	0.098	18.15 (19.88)	21.43 (31.82)	0.006
Had ≥1 ancillary encounter, *n* (%)	3621 (97.50)	1647 (98.45)	1974 (96.72)	<0.001	1818 (97.95)	1803 (97.04)	0.075
Had ≥1 schizophrenia-related ancillary encounter, *n* (%)	2769 (74.56)	1310 (78.30)	1459 (71.48)	<0.001	1439 (77.53)	1330 (71.58)	<0.001
Number of ancillary encounters, mean (SD)	47.53 (56.10)	63.95 (67.29)	34.07 (40.18)	<0.001	59.91 (64.53)	35.17 (42.74)	<0.001
Number of schizophrenia-related ancillary encounters, mean (SD)	28.23 (49.03)	38.63 (19.71)	60.01 (35.52)	<0.001	36.29 (57.33)	20.18 (37.33)	<0.001

a Psychiatric-related relapse event rates and all-cause and schizophrenia-related healthcare utilization compared among patients adherent (MPR ≥ 80%) and non-adherent (MPR < 80%) and persistent (continuous use defined as no refill gap or refill gap of ≤60 days for SGOA medication) and non-persistent (refill gap for SGOA medication > 60 days or SGOA discontinuation) with SGOAs.

b Psychiatric-related relapse event defined as an inpatient admission or ED visit related to psychiatric conditions and measured during the 12-month postindex period after the first observed SGOA use.

c *P* values based on Student's *t* test for continuous variables and chi-squared or Fisher test for categorical variables.

d Length of stay calculated only among patients with ≥1 hospitalization.

ED, emergency department; MPR, medication possession ratio; SD, standard deviation; SGOA, second-generation oral antipsychotic.

Adherent patients had significantly higher rates of all-cause inpatient stays (84.22% versus 81.19%; *P* *=* 0*.*015) than non-adherent patients. The rate of all-cause inpatient stays did not differ between the two persistence groups. However, patients persistent with the SGOA therapy had lower mean all-cause inpatient stay compared with the non-persistent patients (20.58 (23.12) versus 24.89 (33.96); *P* < 0.001). Adherent/persistent patients were less likely to have an all-cause ED visit during the postindex date period than non-adherent (84.16% versus 89.32%; *P* < 0*.*001) and non-persistent (85.18% versus 88.81%; *P* *=* 0*.*001) patients.

All-cause physician office visits were higher among adherent and persistent groups compared with the non-adherent (21.95 (34.22) versus 12.97 (22.14); *P* < 0.001) and non-persistent patients (21.18 (33.47) versus 12.86 (21.89); *P* < 0.001).

### Covariate-adjusted association between second-generation oral antipsychotics adherence and persistence and psychiatric-related relapse events as well as schizophrenia-related and all-cause utilization

Tables 4 and 5 summarize results from multivariable negative binomial and logistic regression analyses, respectively. The rate of psychiatric-related relapses was 10% lower among adherent than non-adherent patients (IRR: 0.90; 95% confidence interval (CI): 0.86–0.94; *P* < 0*.*001). Similarly, the rate of psychiatric-related relapse event was 12% lower among persistent than non-persistent patients (IRR: 0.88; 95% CI: 0.84–0.92; *P* < 0*.*001) (Table 4). Compared with non-persistent patients, persistent patients had a significantly lower rate of schizophrenia-related inpatient admissions (IRR: 0.85; 95% CI: 0.77–0.93; *P* < 0*.*001). Compared with non-adherent and non-persistent patients, adherent (OR: 0.83; 95% CI: 0.71–0.96; *P* *=* 0*.*011) and persistent (OR: 0.76; 95% CI: 0.66–0.88; *P* < 0*.*001) patients had significantly lower likelihood of a schizophrenia-related inpatient admission during the postindex date period (Table 5). Compared with non-adherent and non-persistent patients, patients who were adherent (IRR: 0.79; 95% CI: 0.69–0.90; *P* < 0*.*001) and persistent (IRR: 0.81; 95% CI: 0.71–0.92; *P* < 0*.*001) had significantly lower rates of schizophrenia-related ED visits.

**Table 4:** Rate of events: negative binomial regression results by adherence and persistence status

	Adherent^a^				Persistent^a^			
		
Event Setting	IRR^b^	95% CIs		*P* value	IRR^b^	95% CIs		*P* value
Number of psychiatric-related relapse events	0.90	0.86	0.94	<0.001	0.88	0.84	0.92	<0.001
Number of schizophrenia-related inpatient admissions	0.94	0.85	1.03	0.186	0.85	0.77	0.93	<0.001
Number of inpatient admissions	0.97	0.91	1.03	0.276	0.87	0.82	0.93	<0.001
Number of schizophrenia-related outpatient visits	1.20	1.00	1.43	0.046	1.15	0.97	1.36	0.118
Number of outpatient visits	1.29	1.19	1.40	<0.001	1.27	1.17	1.38	<0.001
Number of schizophrenia-related ED visits	0.79	0.69	0.90	<0.001	0.81	0.71	0.92	0.002
Number of ED visits	0.73	0.67	0.78	<0.001	0.78	0.73	0.85	<0.001
Number of schizophrenia-related office visits	2.10	1.70	2.58	<0.001	1.89	1.54	2.32	<0.001
Number of office visits	1.47	1.35	1.61	<0.001	1.44	1.32	1.57	<0.001
Number of schizophrenia-related prescription claims	1.99	1.91	2.06	<0.001	1.91	1.84	1.98	<0.001
Number of prescription claims	1.81	1.73	1.88	<0.001	1.74	1.67	1.82	<0.001
Number of schizophrenia-related ancillary care encounters	1.85	1.65	2.09	<0.001	1.65	1.47	1.86	<0.001
Number of ancillary care encounters	1.75	1.63	1.88	<0.001	1.57	1.46	1.68	<0.001
Number of schizophrenia-related total non-pharmacy medical encounters	1.78	1.63	1.94	<0.001	1.59	1.46	1.73	<0.001
Number of total non-pharmacy medical encounters	1.51	1.44	1.59	<0.001	1.41	1.34	1.48	<0.001
Number of schizophrenia-related total medical encounters	1.87	1.76	1.98	<0.001	1.70	1.60	1.80	<0.001
Number of total medical encounters	1.65	1.58	1.71	<0.001	1.55	1.50	1.62	<0.001

a Psychiatric-related relapse event rates and all-cause and schizophrenia-related visits (to the selected service settings) compared among patients adherent (MPR ≥ 80%) and non-adherent (MPR < 80%) to SGOAs and persistent (continuous use defined as no refill gap or refill gap of ≤60 days for SGOA medication) and non-persistent (refill gap for the SGOA medication > 60 days or SGOA discontinuation) with SGOAs.

b IRR based on negative binomial regression model adjusted for age, gender, race/ethnicity, health plan, eligibility status, dose escalation, polypharmacy, mental health substance abuse coverage, Charlson Comorbidity Index score and adherence or persistence.

CI, confidence interval; ED, emergency department; IRR, incident rate ratio; MPR, medication possession ratio; SGOA, second-generation oral antipsychotic.

**Table 5:** Likelihood of event: logistic regression results by adherence and persistence status

	Adherent^a^				Persistent^a^			
		
Event Setting	Odds ratio^b^	95% CIs		*P* value	Odds ratio^b^	95% CIs		*P* value
Schizophrenia-related inpatient admission	0.83	0.71	0.96	0.011	0.76	0.66	0.88	<0.001
Inpatient admission	0.91	0.75	1.11	0.362	0.77	0.64	0.93	0.007
Schizophrenia-related outpatient visit	1.20	1.03	1.40	0.021	1.21	1.04	1.40	<0.001
Outpatient visit	1.72	1.42	2.10	<0.001	1.69	1.40	2.04	<0.001
Schizophrenia-related ED visit	0.86	0.73	1.00	0.054	0.89	0.77	1.04	0.135
ED visit	0.64	0.52	0.79	<0.001	0.74	0.60	0.91	0.005
Schizophrenia-related office visit	1.36	1.17	1.58	<0.001	1.27	1.10	1.48	<0.001
Office visit	1.68	1.33	2.12	<0.001	1.86	1.49	2.33	<0.001
Schizophrenia-related ancillary care encounter	1.31	1.11	1.55	0.002	1.23	1.05	1.45	0.012
Ancillary care encounter	1.74	1.06	2.85	0.029	1.13	0.72	1.77	0.588

a Likelihood of overall and schizophrenia-related visits (to the selected service settings) compared among patients adherent (MPR ≥ 80%) and non-adherent (MPR < 80%) to SGOAs and persistent (continuous use defined as no refill gap or refill gap of ≤60 days for SGOA medication) and non-persistent (refill gap for the SGOA medication > 60 days or SGOA discontinuation) with SGOAs.

b Odds ratio based on logistic regression model adjusted for age, gender, race/ethnicity, health plan, eligibility status, dose escalation, polypharmacy, mental-health substance abuse coverage, Charlson Comorbidity Index score and adherence or persistence.

CI, confidence interval; ED, emergency department; MPR, medication possession ratio; SGOA, second-generation oral antipsychotic.

Schizophrenia patients adherent to SGOA therapy had higher rate of schizophrenia-related outpatient (IRR: 1.20; 95% CI: 1.00–1.43; *P* *=* 0.046) and physician office (IRR: 2.10; 95% CI: 1.70–2.58; *P* < 0.001) visits than the non-adherent patients. The rate of schizophrenia-related physician office visits was higher among persistent patients compared with the non-persistent (IRR: 1.89; 95% CI: 1.54–2.32; *P* < 0.001) patients. However, the rate schizophrenia-related outpatient visits did not differ significantly between the two persistence groups.

Persistent patients had a significantly lower rate of all-cause inpatient admissions (IRR: 0.87; 95% CI: 0.82–0.93; *P* < 0*.*001) and ED visits (IRR: 0.78; 95% CI: 0.73–0.85; *P* < 0*.*001) compared with non-persistent patients. Compared with non-persistent patients, persistent patients had a significantly lower likelihood of all-cause inpatient admission during the 12-month postindex date period (OR: 0.77, 95% CI: 0.64–0.93; *P* *=* 0.007) patients. Similarly, the likelihood of an all-cause ER visit during the 12-month postindex date period was significantly lower among adherent (36%; OR: 0.64; 95% CI: 0.52–0.79; *P* < 0.001) and persistent (26%; OR: 0.74; 95% CI: 0.60–0.91; *P* *=* 0.005) patients compared with non-adherent and non-persistent patients respectively.

Finally, among patients adherent to and persistent with SGOA therapy, the rate of total medical encounters was higher, when compared with non-adherent (IRR: 1.65; 95% CI: 1.58–1.71; *P* < 0.001) and non-persistent (IRR: 1.55; 95% CI: 1.50–1.62; *P* < 0.001) patients (Table 4).

## Discussion

The extent of adherence to and persistence with SGOAs among Medicaid-enrolled schizophrenia patients experiencing ≥2 psychiatric-related relapses was examined. During the postindex period, approximately 45% of patients were adherent to their SGOAs, defined as MPR ≥ 0.80. The adherence rate observed was consistent with findings from other Medicaid claims-based studies of adherence to antipsychotic therapy, which have reported adherence (MPR ≥ 0.80) to SGOAs in the range of 41–43%.[27,28] Approximately 50% of patients were persistent with SGOAs, 23% had ≥1 therapy interruption, and 27% discontinued their SGOAs.

The association between adherence to and persistence with SGOAs, psychiatric-related relapse rates, disease-specific and all-cause healthcare utilization during the postindex period was investigated. Both adherent and persistent patients had lower rates of psychiatric-related relapses. Compared with non-persistence with SGOAs, persistence resulted in a lower likelihood of both schizophrenia-related and all-cause inpatient and ED visits. These findings are consistent with other studies that have assessed the association between antipsychotic adherence and/or persistence and the risk of hospitalization.[9,12,28,29] An example is Weiden *et al*., assessing association between partial compliance and risk of re-hospitalizations among schizophrenia patients enrolled in the California Medicaid programme.[9] The findings of this study indicate that a 30-day gap in antipsychotic medications significantly increased the risk of mental-health-related re-hospitalizations (OR: 2.81; 95% CI: 1.80–4.64) among patients with schizophrenia.[9] Similarly, another study conducted among Medicaid-enrolled schizophrenia patients reported that, compared with patients non-adherent to oral antipsychotic medications (cumulative possession ratio < 80%), adherent patients had a significantly lower rate of psychiatric-related hospitalization (34.9% versus 13.5%; *P* < 0.001).[28] Although our study focused on high-risk patients with schizophrenia experiencing multiple psychiatric-related relapses, the directionality of the association between adherence to and persistence with SGOAs and these events and inpatient and ED use is similar between this study and other published research.[9,12,28,29]

To address the issue of lower medication adherence, strategies such as patient education, pharmacist-based patient counselling, support of family and friends and improved access to care have been suggested as ways to improve therapy adherence among patients with chronic disorders, including schizophrenia.[7,30–34] With respect to antipsychotic therapy, simplifying the therapy regimen by once-daily dosing, or by using long-acting injectable antipsychotic medications, may aid in improving adherence.[7,30,33,35,36] A recent study survey outlines expert consensus on issues associated with medication adherence among patients with serious mental-health disorders including schizophrenia. The survey findings indicate that physicians consider several advantages of long-acting injectables, including immediate recognition of non-adherence by the treatment prescriber, ease of use for the patient, lower risk of relapse.[33] Moreover, previous research suggests that schizophrenia patients had improved adherence to injectable antipsychotics compared with oral antipsychotics.[37–39] For example, the electronic Schizophrenia Treatment Adherence Registry was initiated to conduct international, prospective, observational studies comparing long-term outcomes among schizophrenia patients initiating oral antipsychotics or long-acting injectable risperidone.[39] The findings of this study indicate that among patients initiating long-acting injectable risperidone, the therapy retention rate was 81% at the end of the 2 year period compared with 63% for patients initiating oral antipsychotics.[39] Similarly, another retrospective chart study conducted among patients with schizophrenia reported decreased likelihood of hospitalization and shorter duration of hospitalization among long-acting injectable risperidone users compared with oral antipsychotic users.[37] The current study was restricted to SGOAs; future research should focus on assessing therapy adherence, resource utilization and psychiatric-related relapses among patients with schizophrenia using injectable antipsychotic medications.

Finally, this study observed significantly higher schizophrenia-related hospital outpatient and physician office visits among adherent and persistent patients versus non-adherent and non-persistent patients. It is likely that the frequent physician–patient interactions are associated with improved SGOA adherence and persistence. Studies have reported that continuity of care and frequent patient–physician interactions are associated with improved therapy adherence and lower hospitalization rates.[40–42]

This study is subject to limitations inherent in most retrospective claims data analyses. First, medication refill patterns in administrative data, while reflective of real-world prescription dispensing, may not reveal the true intent or directions of the prescribing physician. Failure to refill a prescription may occur for clinically appropriate reasons, such as medication AEs, adverse drug interactions, medication titration or abnormal laboratory results.[43] In administrative claims-based adherence studies, it is often assumed that medication refilled is medication consumed. However, patients may discard the medications before refill or may stockpile medication for future use.[43] These factors may cause the MPR to overestimate actual adherence. Even with these limitations, administrative claims data remain a reliable and well-accepted source for estimating adherence with chronic-use medications by using validated measures, such as the MPR.[8,16,24,43] If ICD-9-CM diagnosis codes were recorded inaccurately, it may have resulted in some patients being misidentified as having schizophrenia. In order to lower the likelihood of misidentification of schizophrenia patients, this study required that the selected patients had ≥2 diagnosis claims for schizophrenia and had ≥1 claim for a schizophrenia-specific medication (an SGOA). Even with these precautions, the validity of our results depended on the accuracy of recordkeeping among providers submitting claims in the Medicaid database. No data on important clinical and demographic factors, including disease severity, medication AEs, or patient occupational status, education level and income were available; this may have an impact on medication adherence and resource utilization. The outcome measures considered for this included schizophrenia-related healthcare utilization and psychiatric-related relapses. However, for the purposes of this study we did not assess costs associated with these outcomes measures. In future, additional research needs to be conducted to assess the costs savings resulting due to the lower rate of hospitalization and ED visits among patients adherent to SGAO therapy compared with non-adherent patients. Also, we did not estimate indirect cost savings that could be attributed to a lower rate of psychiatric relapse, such as increased likelihood of employability,[44] savings in informal care-giving time and better transition to the community.[45,46] Finally, this analysis was limited to US Medicaid enrollees; therefore, these findings may not be generalizable to individuals enrolled in other federal or commercial health plans or to individuals without health coverage.

## Conclusions

This study reinforces the need among patients with schizophrenia for improved treatment adherence and persistence, which may help lower the rate of psychiatric-related relapse. Prior research suggests that Medicaid is the largest payer of mental-health care in the USA, with schizophrenia being the primary cost driver of mental-health care.[21,47,48] Given the declining Medicaid funding, states are likely to adopt cost-cutting measures that may affect medical care access to the vulnerable mental-health population.[49] Thus, interventions that may aid in lowering the rate of psychiatric-related relapse among Medicaid-enrolled patients with schizophrenia may help lower the direct economic burden exerted by this population on the Medicaid system.

One of the interventions to lower the psychiatric-relapse rate would be to improve adherence and persistence to the prescribed therapy among schizophrenia patients. However, despite publication of numerous studies outlining the association between lower therapy adherence and increased risk of hospitalization, improving adherence and persistence among patients with schizophrenia remains a significant challenge to healthcare providers.[11,12,28,50] Nonetheless, interventions aimed at improving antipsychotic therapy adherence, such as pharmacist-based patient counselling, simplifying dosing regimen and use of once-a-day medications or long-acting injectables, may aid in improving therapy adherence among high-risk patients with schizophrenia experiencing multiple psychiatric-related relapses.

## Declarations

### Conflict of interest

Sudeep Karve and Sean D. Candrilli are employees RTI Health Solutions, North Carolina, USA, an independent contract research organization that has received research funding from Janssen Scientific Affairs, LLC, Titusville, New Jersey, for this study; Jessica Panish and Riad Dirani are employees of Janssen Scientific Affairs, LLC. The publication of this study's results is not contingent on the sponsor's approval or censorship of the manuscript.

### Funding

Funding for this study was provided by Janssen Scientific Affairs, LLC, Titusville, New Jersey, USA.

### Authors’ contributions

Sudeep Karve, Jessica Panish, Riad Dirani and Sean Candrilli were the primary developers of the study design. As principal investigator, Sudeep Karve had full access to all the data in the study and takes responsibility for the integrity of the data and the accuracy of the data analysis. Sudeep Karve and Sean Candrilli led all statistical analyses. Sudeep Karve and Jessica Panish were primarily involved in drafting the manuscript text and in interpreting the findings. Sean Candrilli and Riad Dirani assisted in interpreting the study findings and drafting the manuscript text. They also served as the primary reviewers of the manuscript text. All Authors state that they had complete access to the study data that support the publication.
